# Measuring Ergonomic Risk in Operating Surgeons by Using Wearable Technology

**DOI:** 10.1001/jamasurg.2019.6384

**Published:** 2020-03-11

**Authors:** Andrew J. Meltzer, M. Susan Hallbeck, Melissa M. Morrow, Bethany R. Lowndes, Victor J. Davila, William M. Stone, Samuel R. Money

**Affiliations:** 1 Department of Surgery, Mayo Clinic, Phoenix, Arizona; 2 Department of Health Sciences Research, Mayo Clinic, Rochester, Minnesota; 3 Robert D. and Patricia E. Kern Center, Mayo Clinic, Rochester, Minnesota; 4 Department of Surgery, Mayo Clinic, Rochester, Minnesota; 5 Department of Neurological Sciences, University of Nebraska Medical Center, Omaha

## Abstract

This case series study examines the ergonomic risk of surgery using wearable sensor inertial measurement units to monitor the ergonomics of surgeons at work.

The health care workforce faces numerous occupational hazards, leading to rates of injury and absenteeism that exceed those of the construction and manufacturing sectors.^1,2^ To date, efforts to address these problems have focused on improving safety for support staff, nurses, and allied health care personnel. Work-associated pain among surgeons has garnered less attention, despite the implications of practitioner injury and disability on the surgical workforce.^3^

Ergonomists have long recognized the potential hazards facing the surgeon; from the ergonomic standpoint, surgery has been described as “a mess.”^4^^(p1011)^ However, research has suffered from the absence of an objective means to measure surgeons’ ergonomic stress. This study describes the ergonomic risks of surgery using wearable sensor inertial measurement units (IMUs) to monitor the ergonomics of surgeons at work and identifies risk factors for injury.

## Methods

Preoperatively, surgeons had 4 IMUs placed on their head, torso, and upper arms to measure deviations from neutral body position. The IMU sensors measure body-posture angles via the fusion of data from an accelerometer, magnetometer, and gyroscope contained within each sensor. After processing, ergonomic risk was assessed by calculating the percentage of time spent in a specified range of risk categories for each body segment, facilitating stratification into ergonomic risk categories (Figure) using a validated scale.^5,6^ The high-risk classification was based on occupational ergonomic research exposure-response analyses, which have shown clinically significant musculoskeletal disorders and/or discomfort associated with exposure to neck, torso, and arm postures in the high-risk categories. Objectively measured ergonomic risk was compared across procedure categories (eg, open, laparoscopic, and endovascular surgeries), adjunctive equipment (eg, loupes, headlight, lead apron), as well as surgeon characteristics (eg, including self-reported case complexity and physical discomfort using a preprocedure and postprocedure survey instrument). This study was approved by the Mayo Clinic institutional review board, and oral consent was obtained from all participants. The α level was set at 5%. Statistical analyses were performed using SPSS, version 26 (IBM), and statistical significance was set at a 2-tailed *P *< .05.

**Figure.**
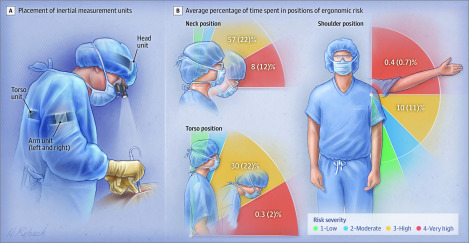
Measurement of Ergonomic Risk of the Neck, Torso, and Shoulder of Surgeons While Operating High-risk positions (categories 3-4; yellow and orange) for the neck (A), torso (B), and shoulder (C), with the mean (SD) percentage of time for categories 3 and 4 included.

## Results

Fifty-three surgeons (19 women [35.8%]; mean [SD] age, 45 [11] years) representing 12 surgical specialties underwent continuous IMU recording during 115 cases (Table). Overall, surgeons spent 65% of procedure time in high-risk neck positions (Table; Figure, A). High-risk positions for the torso and shoulders were observed during 30% and 11% of the minutes of procedure time, respectively. The highest postural neck risk for surgeons was during open vs laparoscopic procedures (adjusted odds ratio, 31.1 [95% CI, 8.47-114.41]; *P* < .001).

**Table.** High-Risk^a^ Position Time by Surgical Specialty

Surgical Specialty	Total Cases, No. (%)	Mean (SD) Time in High-Risk Position, %			
		Neck	Torso	Right Shoulder	Left Shoulder
Cardiothoracic	4 (3.5)	52.7 (32.0)	20.6 (26.9)	9.2 (9.2)	6.7 (7.1)
Colorectal	11 (9.6)	50.9 (18.5)	26.5 (17.5)	6.3 (3.2)	4.1 (4.8)
General	25 (21.7)	59.6 (28.8)	28.7 (22.5)	9 (15.1)	7.2 (12.5)
Gynecologic	10 (8.7)	61 (29.7)	32.1 (27.7)	13.2 (10.4)	5.4 (4.1)
Head and neck	6 (4.4)	77.6 (19.1)	25.7 (5.5)	9.3 (4.9)	6.1 (6.1)
Hepatobiliary	8 (7)	78.4 (27.1)	34 (15.6)	15.6 (17.4)	6.7 (11.9)
Neurosurgery	5 (4.4)	60.4 (27.7)	47.2 (35.0)	14.9 (10.8)	4.6 (4.4)
Orthopedic	7 (6.1)	69.1 (11.7)	50 (25.7)	13 (7.1)	8.2 (2.2)
Plastic	3 (2.6)	85.4 (9.8)	31.4 (25.1)	9.7 (4.8)	4.7 (0.8)
Urology	11 (9.6)	56.3 (23.1)	21 (25.0)	10.8 (14.7)	6.4 (5.1)
Vascular	25 (21.7)	71.5 (25.2)	28.2 (21.2)	7.3 (7.6)	5.4 (9.0)

^a^ Categories 3 and 4.

The use of surgical loupes and headlamps were both independently associated with increased time in ergonomically unfavorable neck positions (mean [SD] with loupes, 85.2% [14.5%]; *P* < .001; without loupes, 58.1% [25.7%]; *t*_110_ = 5.11; *P* < .007; with headlamps, 79.9% [15.7%]; without headlamps, 62.2% [26.7%]; *t*_110_ = 2.42; *P* = .02). Risk factors for surgeon-reported pain included longer case length (*F*_1,105_ = 7.61; *P* < .001), increased years in practice (*F*_2,84_ = 4.42; *P* = .02), and use of loupes (*t*_105_ = 5.42; *P* < .001) and headlights (*t*_105_ = 2.75; *P* = .01). Surgeon self-reported pain was associated with ergonomic risk (*F*_1,10_ = 6.43; *P* < .01).

## Conclusions

The physical demands of performing surgery are real. A surgeon’s cervical spine, in particular, is at unacceptably elevated risk during many procedures. Poor ergonomics are a cause of chronic pain and disability for many surgeons, reducing career longevity and threatening the public’s access to surgical care.^3,4^ This study demonstrates the utility of wearable technology as a means to assess surgeons’ intraoperative ergonomics and postural behavior, providing an evidence base and method for future objective research in this area. The limitations of the study include the sample size and selection bias inherent in selecting surgeon participants. Hopefully, this Research Letter encourages further investigation and induces surgeons to be mindful of their intraoperative ergonomics.
